# Supplementary material for: Both partners’ negative emotion drives aggression during couples’ conflict
Source: Commun Psychol. 2024 Aug 7;2:73. doi: 10.1038/s44271-024-00122-4 (PMC11331989; doi:10.1038/s44271-024-00122-4)
Supplement: Supplementary file 2 — Supplemental Information [file 44271_2024_122_MOESM2_ESM.pdf]

1. Tables

1.1. Table SI1

Table SI1. EMFACS Codes used to identify positive and negative emotion

Emotion	AU Combination
Happiness	6 + 12[59,60]
Sadness	1 + 4 + **15**[59,60]
Surprise	1 + 2 + 5 + 26[59,60]
Fear	1 + 2 + 4 + 5 + 7 + 20 + 26[59,60]
Anger	4 + 5 + 7 + 23[59,60]
Disgust	9 + 15 + **17**[59,60]

Note. **AU:** Action Unit. Bolded Action Units indicate a modification from reference materials. AU5 replaced with 15 in sadness to capture only affects displayed on both the upper and lower face (as per EMFACS8 (1998) coding recommendations). AU16 replaced with 17 in disgust as OpenFace is unable to reliably detect AU16 [33].

1.2. Table SI2

Table SI2. Shapiro-Wilk Tests of Normality

Panel	Data	W	P-value
Figure 2			
b	Positive Affect	0.92	**<2e-16**
	Negative Affect	0.65	**<2e-16**
Figure 3			
a	Positive Affect	0.92	**3e-4**
	Negative Affect	0.83	**9e-8**
b	Immediate Response	0.83	**9e-8**
	Forced Break	0.91	**2e-8**
c	Immediate Response	0.91	**2e-5**
	Forced Break	0.89	**5e-11**
d	Immediate Response	0.91	**4e-12**
	Forced Break	0.89	**<2e-16**
e	Time 1	0.86	**7e-11**
	Time 2	0.71	**<2e-16**
f	Immediate Response	0.89	**0.002**
	Forced Break	0.93	**5e-7**
Figure 4			
NA	Neither	0.90	**3e-10**
	Loser	0.90	**9e-5**
	Winner	0.87	**8e-5**
	Both	0.76	**1e-5**
Figure 8			
a	Forced Break	0.91	**9e-7**
b	Immediate Response	0.86	**<2e-16**
	Forced Break	0.88	**<2e-16**

1.3. Table SI3

Table SI3. Summary Statistics for Extended Analysis of Conditions

Variable/Comparison	Mean	SD
Figure C1		
Mean Blast in 0 Second Break Condition		
High Negative	5.58	2.26
Low Negative	3.59	2.15
High Positive	4.93	2.33
Mean Blast in 5 Second Break Condition		
High Negative	3.65	2.20
Low Negative	3.75	2.16
High Positive	4.52	2.47
Mean Blast in 10 Second Break Condition		
High Negative	3.80	2.50
Low Negative	3.67	2.35
High Positive	4.89	2.63
Mean Blast in 15 Second Break Condition		
High Negative	3.66	2.04
Low Negative	3.46	3.39
High Positive	4.69	2.67
Negative Affect Levels at Winner Reveal		
0s Break	0.07	0.11
5s Break	0.09	0.17
10s Break	0.10	0.21
15s Break	0.08	0.19
Negative Affect Levels at Blast Initiation		
0s Break	0.21	0.27
5s Break	0.11	0.19
10s Break	012	0.19
15s Break	0.07	0.16

1.1. Table SI4

Table SI4. Full Statistics for Extended Analysis of Conditions

Sample	Test	Panel	n	k	Effect Size	P-value	Bayes Factor	95% CI ↓	95% CI ↑	df	Stat
Figure SI1											
Winners with high negativity	Wilcoxon Signed-Rank Test	A 0-5	47	146	0.38	**5e-6**	6012	1.00	3.00	NA	3823
		A 0-10	35	113	0.33	**4e-4**	52.93	0.99	2.99	NA	2052
		A 0-15	37	113	0.37	**8e-5**	295.2	1.00	3.00	NA	2128
		A 5-10	44	115	0.02	0.86	0.22	-0.99	0.99	NA	1488
		A 5-15	46	115	0.01	0.89	0.21	-0.99	0.00	NA	1495
		A 10-15	34	82	0.01	0.92	0.24	-0.99	1.00	NA	852
		B	81	228	0.35	**1e-7**	2e+5	1.00	2.99	NA	8002

1.1. Table SI5

Table SI5. Full Statistics for Recreation of Analysis (Figure 4) with Expanded Conditions

Panel	n	k	Effect Size	P-value	Bayes Factor	95% CI ↓	95% CI ↑	Stat
5 Second Condition								
B	47	146	0.38	**4e-6**	6012	1.00	3.00	3823
C	43	167	0.01	0.95	0.17	-0.99	0.99	3256
D	55	681	0.04	0.32	0.13	-4e-5	3e-5	5e+4
E	24	72	0.51	**7e-10**	1e+6	-0.29	-0.19	4134
F	24	72	0.22	**4e-4**	1e+22	0.11	0.32	1746
10 Second Condition								
B	35	113	0.33	**4e-4**	52.92	0.99	2.99	2052
C	31	92	0.14	0.18	0.55	-2.00	8e-8	756
D	46	577	1e-3	0.97	0.10	-4e-5	1e-7	4e+4
E	16	47	0.65	**4e-10**	1e+8	-0.49	-0.32	1928
F	16	47	0.18	**1e+7**	1e+14	0.21	0.49	1327
15 Second Condition								
B	37	113	0.37	**8e-5**	295.3	1.00	3.00	2128
C	32	102	0.04	0.68	0.24	-1.00	0.99	1169
D	49	614	0.06	0.16	0.12	-4e-5	9e-1	5e+4
E	21	49	0.80	**2e-15**	2e+9	-0.37	-0.27	2315
F	21	49	0.23	**2e-7**	2e+20	0.19	0.45	1365

2. Figures

2.1. Figure SI1

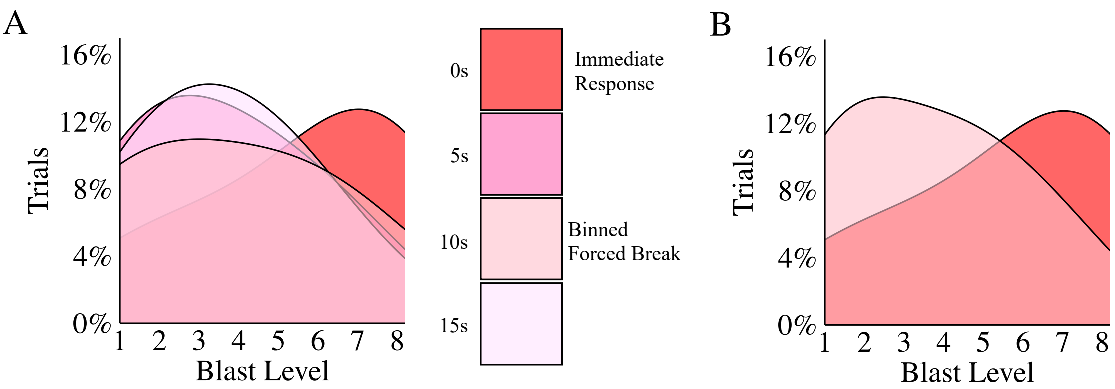

Forced Breaks Reduced Aggression Regardless of Duration. Panel a. Within the forced break conditions, the precise length of break imposed appears uninformative. **Panel b.** Because of the lack of differentiation between the forced break conditions, we collapsed the five, 10, and 15 second conditions into one combined "forced break" condition for analysis of the differences in behaviour between the immediate response and the forced break conditions.
