# Supplementary material for: Both partners’ negative emotion drives aggression during couples’ conflict
Source: Commun Psychol. 2024 Aug 7;2:73. doi: 10.1038/s44271-024-00122-4 (PMC11331989; doi:10.1038/s44271-024-00122-4)
Supplement: Supplementary file 3 — Reporting Summary [file 44271_2024_122_MOESM3_ESM.pdf]

nature portfolio

Corresponding author(s): Annah McCurry

Last updated by author(s): May 16, 2024

Reporting Summary

Nature Portfolio wishes to improve the reproducibility of the work that we publish. This form provides structure for consistency and transparency in reporting. For further information on Nature Portfolio policies, see our Editorial Policies and the Editorial Policy Checklist.

Statistics

For all statistical analyses, confirm that the following items are present in the figure legend, table legend, main text, or Methods section.

n/a	Confirmed	
☐	☒	The exact sample size (n) for each experimental group/condition, given as a discrete number and unit of measurement
☐	☒	A statement on whether measurements were taken from distinct samples or whether the same sample was measured repeatedly
☐	☒	The statistical test(s) used AND whether they are one- or two-sided<br>*Only common tests should be described solely by name; describe more complex techniques in the Methods section.*
☐	☒	A description of all covariates tested
☐	☒	A description of any assumptions or corrections, such as tests of normality and adjustment for multiple comparisons
☐	☒	A full description of the statistical parameters including central tendency (e.g. means) or other basic estimates (e.g. regression coefficient) AND variation (e.g. standard deviation) or associated estimates of uncertainty (e.g. confidence intervals)
☐	☒	For null hypothesis testing, the test statistic (e.g. F, t, r) with confidence intervals, effect sizes, degrees of freedom and P value noted<br>*Give P values as exact values whenever suitable.*
☐	☒	For Bayesian analysis, information on the choice of priors and Markov chain Monte Carlo settings
☐	☒	For hierarchical and complex designs, identification of the appropriate level for tests and full reporting of outcomes
☐	☒	Estimates of effect sizes (e.g. Cohen's d, Pearson's r), indicating how they were calculated

Our web collection on statistics for biologists contains articles on many of the points above.

Software and code

Policy information about availability of computer code

Data collection	We used a custom Python (version 3) program to run our experiment. We used Quartics to administer the post-experimental questionnaire.
Data analysis	We used R (version 4.3) to analyze all data.

For manuscripts utilizing custom algorithms or software that are central to the research but not yet described in published literature, software must be made available to editors and reviewers. We strongly encourage code deposition in a community repository (e.g. GitHub). See the Nature Portfolio guidelines for submitting code & software for further information.

Data

Policy information about availability of data

All manuscripts must include a data availability statement. This statement should provide the following information, where applicable:

- Accession codes, unique identifiers, or web links for publicly available datasets
- A description of any restrictions on data availability
- For clinical datasets or third party data, please ensure that the statement adheres to our policy

De-identified data available at DOI: 10.17630/a4fd6eb0-34e3-4999-8dd3-b407a5720146. Raw video/audio data is not available to protect participant anonymity.

Human research participants

Policy information about studies involving human research participants and Sex and Gender in Research.

Reporting on sex and gender	A total of 162 participants (89 women, 4 non-binary participant; assessed by the item "what is your gender?" in the post-experiment questionnaire)
Population characteristics	We recorded age, gender, if gender is different from sex assigned at birth, occupation, ethnicity, and information about co inhabitation with ones partner.
Recruitment	Participants were recruited from a participant pool at the University of St Andrews in Scotland. Participants were romantic couples (of any sexual orientation) who participated together, and each partner was given £12.50 in compensation.
Ethics oversight	This study was originally approved by the School of Psychology and Neuroscience Ethics Committee in December 2022 (approval code PS16636; revised for follow-up in January 2024) before data collection commenced. The study was not pre-registered.

Note that full information on the approval of the study protocol must also be provided in the manuscript.

Field-specific reporting

Please select the one below that is the best fit for your research. If you are not sure, read the appropriate sections before making your selection.

☐ Life sciences ☒ Behavioural & social sciences ☐ Ecological, evolutionary & environmental sciences

For a reference copy of the document with all sections, see nature.com/documents/nr-reporting-summary-flat.pdf

Behavioural & social sciences study design

All studies must disclose on these points even when the disclosure is negative.

Study description	Mixed-method design.
Research sample	Primary study: Participants' mean age was 21.1 years (SD= 3.3) with a minimum of 18 and maximum of 39. Most participants were full time students (72.8%) and white (70.3%). Most participants were not cohabiting (66.1%), and the average duration of a relationship was roughly eight months.<br><br>Follow-on study: Participants' mean age was 21.9 years (SD= 7.4) with a minimum of 18 and maximum of 62. Most participants were full time students (80.0%) and white (83.3%). Most participants were not cohabiting (70.0%), and the average duration of a relationship was roughly fifteen months.
Sampling strategy	We used convenience sampling. Participants were recruited by poster and online through through the University of St Andrews internal Memo system (which send weekly recruitment emails.
Data collection	The researcher was not blind during experimentation and no-one was present except for the researcher and the participants. Consent was recorded on pen and paper. All other data was recorded digitally on computers or phones. Audio/video was recorded using an Insta360 camera.
Timing	Collection for the Primary study ran from November 2023 to December 2023. Collection for the follow-up ran from February 2024 to March 2024
Data exclusions	There were no data exclusions in the primary study. In the follow on study, the CRTT data from one couple (2 participants) did not write correctly out of python (meaning their primary data file was corrupt) and so their game data could not be used.
Non-participation	In the primary study, one participant withdrew data.
Randomization	Participants were randomly assigned to conditions (of which there were two) using a digital coin flip.

Reporting for specific materials, systems and methods

We require information from authors about some types of materials, experimental systems and methods used in many studies. Here, indicate whether each material, system or method listed is relevant to your study. If you are not sure if a list item applies to your research, read the appropriate section before selecting a response.

Materials & experimental systems

n/a	Involved in the study	
☒	☐	Antibodies
☒	☐	Eukaryotic cell lines
☒	☐	Palaeontology and archaeology
☒	☐	Animals and other organisms
☒	☐	Clinical data
☒	☐	Dual use research of concern

Methods

n/a	Involved in the study	
☒	☐	ChIP-seq
☒	☐	Flow cytometry
☒	☐	MRI-based neuroimaging
